# Whole Genome Analysis of SNV and Indel Polymorphism in Common Marmosets (*Callithrix jacchus*)

**DOI:** 10.3390/genes14122185

**Published:** 2023-12-07

**Authors:** R. Alan Harris, Muthuswamy Raveendran, Wes Warren, Hillier W. LaDeana, Chad Tomlinson, Tina Graves-Lindsay, Richard E. Green, Jenna K. Schmidt, Julia C. Colwell, Allison T. Makulec, Shelley A. Cole, Ian H. Cheeseman, Corinna N. Ross, Saverio Capuano, Evan E. Eichler, Jon E. Levine, Jeffrey Rogers

**Affiliations:** 1 Human Genome Sequencing Center and Department of Molecular and Human Genetics, Baylor College of Medicine, Houston, TX 77030, USA; rharris1@bcm.edu (R.A.H.); raveendr@bcm.edu (M.R.); 2 Bond Life Sciences Center, University of Missouri, Columbia, MO 65211, USA; warrenwc@missouri.edu; 3 Department of Genome Sciences, University of Washington School of Medicine, Seattle, WA 98104, USA; lhillier@uw.edu (H.W.L.); ee3@uw.edu (E.E.E.); 4 McDonnell Genome Institute, Washington University, St. Louis, MO 63108, USA; ctomlins@wustl.edu (C.T.); tgraves@wustl.edu (T.G.-L.); 5 Department of Biomolecular Engineering, University of California, Santa Cruz, CA 95064, USA; regreen@ucsc.edu; 6 Wisconsin National Primate Research Center, University of Wisconsin, Madison, WI 53715, USA; jkropp@wisc.edu (J.K.S.); gambardella2@wisc.edu (J.C.C.); makulec@wisc.edu (A.T.M.); capuano@primate.wisc.edu (S.C.III); levine@primate.wisc.edu (J.E.L.); 7 Southwest National Primate Research Center, Texas Biomedical Research Institute, San Antonio, TX 78227, USA; scole@txbiomed.org (S.A.C.); ianc@txbiomed.org (I.H.C.); cross@txbiomed.org (C.N.R.); 8 Howard Hughes Medical Institute, University of Washington, Seattle, WA 98195, USA

**Keywords:** marmoset, single nucleotide variants, twinning, platyrrhine, reference genome, genome assembly

## Abstract

The common marmoset (*Callithrix jacchus*) is one of the most widely used nonhuman primate models of human disease. Owing to limitations in sequencing technology, early genome assemblies of this species using short-read sequencing suffered from gaps. In addition, the genetic diversity of the species has not yet been adequately explored. Using long-read genome sequencing and expert annotation, we generated a high-quality genome resource creating a 2.898 Gb marmoset genome in which most of the euchromatin portion is assembled contiguously (contig N50 = 25.23 Mbp, scaffold N50 = 98.2 Mbp). We then performed whole genome sequencing on 84 marmosets sampling the genetic diversity from several marmoset research centers. We identified a total of 19.1 million single nucleotide variants (SNVs), of which 11.9 million can be reliably mapped to orthologous locations in the human genome. We also observed 2.8 million small insertion/deletion variants. This dataset includes an average of 5.4 million SNVs per marmoset individual and a total of 74,088 missense variants in protein-coding genes. Of the 4956 variants orthologous to human ClinVar SNVs (present in the same annotated gene and with the same functional consequence in marmoset and human), 27 have a clinical significance of pathogenic and/or likely pathogenic. This important marmoset genomic resource will help guide genetic analyses of natural variation, the discovery of spontaneous functional variation relevant to human disease models, and the development of genetically engineered marmoset disease models.

## 1. Introduction

The common marmoset (*Callithrix jacchus*) is a South American platyrrhine monkey that has become increasingly utilized as a model organism for biomedical research due to its close genetic and physiological similarity to humans. Marmosets, together with other members of the subfamily Callitrichinae (callitrichines), are small in size with the average weight of a captive adult common marmoset being 350 to 450 g [1]. Common marmosets and other callitrichines also have a high reproductive rate due to routine dizygotic twinning, a postpartum estrus, and a short gestation period of 5 months [2,3]. This suite of characteristics raises questions regarding the genetic basis of this small body size and unusually high reproductive rate compared with other primates. This species also has a relatively long lifespan compared with other model organisms such as mice, averaging 5 to 7 years with a maximum of 16.5 years [4]. This combination of factors makes them useful for studying many human diseases and disorders including Parkinson’s disease [5], and infectious diseases such as Zika virus [6] and COVID-19 [7]. For a recent review of the biology, behavior, biogeography, and ecology of common marmosets, see Malukiewicz et al. [8].

Marmosets are valuable models for both neurodevelopmental disorders [9] and Alzheimer’s disease [10] research due to their shared similarities with humans in brain structure and function, which makes them distinct from rodents. Their relatively long lifespan facilitates the study of age-related diseases, such as Alzheimer’s, while their advanced cognitive abilities allow for investigations into complex functions affected by both disorders [9]. Additionally, their suitability for imaging techniques and sophisticated behavioral monitoring makes them useful for investigating the underlying mechanisms and potential interventions for both neurodevelopmental disorders and Alzheimer’s disease.

A reference genome for the common marmoset was first published in 2014 [11], and since then, several genome assemblies have been generated, each with improvements in contiguity and completeness [12,13]. The availability of a high-quality reference genome is crucial for interpreting functional genomics experiments, identifying candidate genes, and investigating the evolutionary history of the species. DNA sequence variation within the marmoset nuclear genome has not been extensively characterized. Identifying single nucleotide variants (SNVs) and small insertions/deletions (indels) in marmosets is an important step in understanding the genetic diversity and structure of different marmoset populations. Genetic variation within a species is also an important consideration in biomedical research, as it can influence disease susceptibility, drug efficacy, and toxicity. SNVs are the most common type of genetic variation in the genome and are widely used as genetic markers for population studies, genetic mapping, and genotype–phenotype association analysis. Studies of natural genetic variation in other primate species have identified spontaneous models of human genetic disease [14,15]. Surveys of genetic variation among marmosets provide the opportunity to discover mutations in marmoset genes that are orthologous to genes known to cause disease in humans (see the ClinVar [16] and OMIM (omim.org) databases).

In this study, we present a new genome assembly for the common marmoset and identify SNVs and indels using deep (>30x) whole genome sequencing data from 84 marmosets from four distinct sources. This improved assembly and initial variant catalog will provide a valuable resource for researchers using this model organism for biomedical research, ultimately leading to improved human health outcomes. The assembly will also be useful in analyses of genome evolution, comparative genomics, and the genetic composition of wild marmoset populations.

## 2. Materials and Methods

### 2.1. Marmoset Genome Assembly

We generated a new assembly of the marmoset genome using a series of iterative processes similar to that described for the rhesus macaque genome in Warren et al. [17]. In brief, DNA from a female common marmoset (*C. jacchus*) was derived from a cell line (cj1700) that was determined to be of normal karyotype. Single-molecule, real-time (SMRT) sequences were generated using the Pacific Biosciences (PacBio) Sequel instrument (V2 chemistry) to approximately 70x genome coverage using the continuous long-read sequencing platform. All quality-filtered SMRT sequences were assembled using FALCON-Integrate v1.7.5 and then error-corrected using the Arrow correction module. Polishing of the assembly for mostly insertion and deletion base errors was conducted via alignment of 40x sequence coverage (same female individual) of Illumina short-read data (100 bp length) using first the Pilon algorithm [18] followed by a modified Free–Bayes protocol, the latter as described in Kronenberg et al. [19]. Error-corrected contigs were iteratively scaffolded with physical and proximity mapping data, BioNano Genomics, and HiC, respectively, again from the same reference individual. Chromosomal-scale scaffolds were then checked for inconsistencies with alignments to the prior version of the marmoset genome, Callithrix jacchus 3.2, and the human genome (GRChg38.p12), both using BLAT. We also made comparisons to available marmoset FISH maps (http://www.biologia.uniba.it/marmoset/, accessed on 28 November 2023). Any discrepancies discovered were manually evaluated and corrected if necessary. All assembly sequence sources are available under NCBI BioProject PRJNA566173.

### 2.2. DNA Sample Preparation for Studies of Within-Species Variation

DNA was extracted from a total of 84 common marmosets consisting of fibroblast samples from the Wisconsin National Primate Research Center (WNPRC) (*n* = 61) and hair follicle samples from the Southwest National Primate Research Center (SNPRC) (*n* = 23) (Table S1). Among those 84 marmosets, 16 originated from the now-defunct New England Primate Research Center (NEPRC) and 18 originated from the Defence Science and Technology Laboratory, United Kingdom. The animals obtained from the UK are now part of the WNPRC colony.

We note that most marmosets exhibit significant levels of hematopoietic chimerism [20]. Early in embryonic development, the placental circulation systems of two dizygotic co-twin embryos fuse, allowing the exchange of stem cells. This exchange results in lifelong presence in a given marmoset of hematopoietic cell lineages (e.g., bone marrow, leukocytes, and other cell types) that originated in their co-twin and carry that co-twin’s genome [20]. This is the reason sequencing of the germline genomic DNA of these animals requires the use of fibroblasts, hair follicles, or other DNA sources that do not include cells from an individual’s co-twin.

### 2.3. Fibroblast Derivation from Ear Biopsies

All procedures concerning the WNPRC animals were performed in accordance with the University of Wisconsin-Madison Institutional Animal Care and Use Committee-approved protocol and the WNPRC-approved standard operating procedures. For pinna biopsies, marmosets were anesthetized with intramuscular alfaxalone (8 mg/kg). Once anesthetized, the animal’s lower region of the left ear pinna was shaved, and the skin was scrubbed with povidone–iodine followed by isopropyl alcohol. A triangular piece of tissue measuring 2 mm on each side was then excised from the peripheral lower edge of the pinna. Oral meloxicam (0.2 mg/kg) or oral acetaminophen (3.2 mg) was administered post biopsy to ameliorate pain and inflammation.

The pinna tissue from WNPRC animals was immediately transferred to a Petri dish containing 500 µL of marmoset biopsy medium consisting of DMEM/F12 (Thermo Scientific, Waltham, MA, USA, cat No.: SH30023) and 0.5% Pen-Strep (Thermo Scientific, Waltham, MA, USA, cat No.: 15140122). Samples were processed by lightly removing any remaining hair and cutting them into 1 mm diameter pieces. Tissue pieces were either placed directly into a well of a 6-well plate pre-coated with 0.1% gelatin (Sigma Aldrich, St. Louis, MO, USA, cat No.: ES006B) or placed into a 1.5 mL Eppendorf tube containing 500 µL of digestion medium consisting of 80% high-glucose DMEM (Thermo Scientific, Waltham, MA, USA, cat No.: 11960044), 20% fetal bovine serum (Peak Serum Inc., Wellington, FL, USA, cat No.: PSFB4), 0.25% Collagenase type I (Worthington Biochemical, Lakewood, CA, USA, cat No.: LS004194), 0.05% DNAse I (Sigma-Aldrich, cat No.: DN25), and 1.0% Pen-Strep (Thermo Scientific, Waltham, MA, USA, cat No.: 15140122). Tissue samples in digestion media were incubated at 37 °C for three hours and then vortexed for 20 s to allow for separation of the epidermis and disintegration of the dermis. The biopsy pieces were removed from the digestion media and plated into individual wells of a 6-well plate pre-coated with 0.1% gelatin containing 500 µL of marmoset fibroblast growth medium (MFGM) consisting of 80% dermal fibroblast growth medium (Thermo Scientific, Waltham, MA, USA, cat No.: 50306256), 20% Fetal Bovine Serum, 0.1% FGF2, and 1.0% antibiotic–antimycotic (Thermo Scientific, Waltham, MA, USA, cat No.: 15240062). A cell pellet was obtained upon centrifugation at 1000 rpm for 5 min. The cell pellet was resuspended in MFGM and then distributed to the 6-well plate containing tissue biopsies and incubated at 37 °C in 5% CO_2_. The MFGM was exchanged every other day and fibroblast growth was monitored.

Cells were passaged by aspirating the media, rinsing the vessel with PBS (Fisher Scientific, Waltham, MA, USA, cat No.: BP3991), and then incubating in 0.25% Trypsin (Gibco, Waltham, MA, USA, cat No.: 15090046) for 5–10 min at 37 °C. Cells were counted and plated at a density of 150,000 per well of a 6-well plate or ~1 × 10^6^ cells per T75 flask. Approximately 1–1.5 million cells were either stored at −80 °C for future DNA isolation or cryopreserved in CELLBANKER^®^ 1 (Thermo Scientific, Waltham, MA, USA, cat No.: NC0315222) and stored in liquid nitrogen. The Baylor College of Medicine Human Genome Sequencing Center (BCM-HGSC) received ~1 million fibroblast cells per marmoset sample and used the Gentra Puregene kit to isolate high-quality genomic DNA. The DNA samples were processed for library preparation after quantity and quality checks by PicoGreen and gel imaging.

### 2.4. Collection of Hair Follicles and Isolation of DNA from SNPRC Marmosets

Hair and follicles were collected at the time of a physical exam or euthanasia. A body site free of scent-marking secretions was chosen and a clump of 50–100 hairs was grasped with clean forceps and pulled to remove hair with follicles intact. The clump was placed follicle end first into a sterile screw cap tube and frozen at −80 °C. For DNA isolation, the hair and follicles were rinsed with 2 mL of phosphate-buffered saline (2x). DNA was purified using the QIAmp DNA Mini kit (Qiagen, Hilden, Germany) following the manufacturer’s protocol for tissues, including the optional RNase step.

### 2.5. WGS Library Preparation

Whole genome sequencing data were generated for 84 marmoset samples at the BCM-HGSC using established library preparation and sequencing methods. Libraries were prepared using KAPA Hyper PCR-free library reagents (KK8505, KAPA Biosystems Inc. Wilmington, DE, USA) on Beckman robotic workstations (Biomek FX and FXp models). Briefly, DNA (750 ng) was sheared into fragments of approximately 200–600 bp using the Covaris E220 system (96 well format, Covaris, Inc., Woburn, MA, USA) followed by purification of the fragmented DNA using AMPure XP beads. A double-size selection step was employed, with different ratios of AMPure XP beads, to select a narrow size band of sheared DNA molecules for library preparation. DNA end-repair and 3′-adenylation were then performed in the same reaction followed by ligation of the Illumina unique dual barcode adapters (Cat# 20022370) to create PCR-free libraries. The library was evaluated using the Fragment Analyzer (Advanced Analytical Technologies, Inc., Ames, IA, USA) to assess library insert size and the presence of remaining adapter dimers. This was followed by a qPCR assay with the KAPA Library Quantification Kit (KK4835) using their SYBR^®^ FAST qPCR Master Mix (KAPA Biosystems Inc. Wilmington, DE, USA) to estimate the size and quantification.

### 2.6. Illumina Whole Genome Sequencing

The Illumina whole genome libraries were pooled and sequenced using S4 flow cells and the NovaSeq SBS reagent kit v1.5 on the NovaSeq 6000 (Illumina, San Diego, CA, USA) instruments according to the manufacturer’s procedures. Briefly, a pool of sequencing libraries (about 250 pM) was loaded onto the S4 flow cell using the XP 4-lane kit. Cluster generation and 2x 150 cycles of sequencing were performed to generate 150 bp paired-end reads with on-instrument base calling. The resulting sequence data were transferred from the instruments for further processing, including mapping, using the HGSC workflow management system HgV (‘Mercury V’, version 17.5) [21]. All sequencing data are available under NCBI BioProject PRJNA955237 (https://www.ncbi.nlm.nih.gov/bioproject/PRJNA955237, accessed on 28 November 2023).

### 2.7. Mapping and Variant Calling

BWA-MEM (v0.7.15) [arXiv:1303.3997v2, accessed on 28 November 2023] was used to map all sequence reads to the Callithrix_jacchus_cj1700_1.1 (GCF_009663435.1) reference genome, which also included the mitochondrial genome (NC_025586.1). To identify reads potentially originating from a single fragment of DNA and mark them in the BAM files, we used Picard MarkDuplicates (v2.6.0). Variants were then called using GATK (v4.2.2.0) [22,23] following best practices and a joint-called VCF file was generated. The hard filters suggested by the developers of GATK (https://gatk.broadinstitute.org/hc/en-us/articles/360035532412?id=11097, accessed on 28 November 2023) were applied to the SNVs and indels and all failing variants were removed. We then used the GATK VariantAnnotator to annotate SNVs applying AlleleBalance. SNVs with an allelic balance for heterozygous calls (ABHet = ref/(ref + alt)) ABHet < 0.2 or ABHet > 0.8 were removed. Similar allele balance filtering was performed for indels using an in-house script. Indels longer than 6 bp were also removed. The Ensembl Variant Effect Predictor (VEP) (v98) [24] was used to annotate the variants in the VCF file based on NCBI RefSeq gene annotations. The average sequencing coverage used for SNV genotype calls in the samples was 34.21x.

### 2.8. PCA and ADMIXTURE Analyses

We performed principal component analysis (PCA) using SNV genotypes from all 84 genomes. Autosomal SNVs, excluding unplaced scaffolds, were filtered with PLINK (v1.90) [25] for missing call rates >0.05 (--geno 0.05) and minor allele frequency <0.1 (--maf 0.1) resulting in a dataset of 7,605,855 SNVs. PLINK --pca was performed on this dataset to generate eigenvectors, and principal components 1 and 2 were plotted using the R plot function. LD pruning was performed to remove related SNVs prior to analysis with ADMIXTURE or calculation of inbreeding coefficients and runs of homozygosity (ROH) for genome diversity analyses. We performed LD pruning with the function PLINK --indep 50 5 2 reducing the number of segregating SNVs to 2,542,325. We applied ADMIXTURE (v1.30) [26] to assess population structure in the marmoset research colonies. The ADMIXTURE cross-validation procedure identified the best-fitting model as K = 3 (CV error = 0.26717).

### 2.9. Genome Diversity

We calculated diversity statistics for individual marmosets and examined them in the context of the populations outlined in the PCA plot. We calculated genome-wide heterozygosity as (autosomal heterozygous SNV calls)/(ungapped autosomal assembly length − missing SNV calls). We also used PLINK (v1.90) to calculate estimates of inbreeding coefficients (F) (plink --het) and runs of homozygosity (ROH) (plink --homozyg) for marmosets across the defined research populations. We performed Welch two-sample t-tests comparing heterozygosity and inbreeding coefficients of WNPRC marmosets to marmosets from the other colonies, excluding crosses, and Human 1000 Genomes data using the R *t*-test function.

### 2.10. Variant Orthologs in Human and Pathogenicity Predictions

In order to assess the impact of variants in orthologous human genes, we projected the marmoset variants onto the human genome (GRCh38). This was carried out by using the Picard (v2.18.14) LiftoverVcf tool with LIFTOVER_MIN_MATCH = 0.95 and RECOVER_SWAPPED_REF_ALT = true. This means that if a marmoset ALT allele is the REF allele in human, then the REF and ALT alleles will be swapped, otherwise the SNV will be removed. Picard LiftoverVcf was performed reciprocally requiring that human sites must lift over back to their original position on marmoset in order to be retained. A total of 11,872,309 SNVs (62.04% of all marmoset SNVs) reciprocally lifted over to human, including 3,921,721 SNVs where the REF and ALT were swapped in human relative to marmoset. A total of 1,020,622 indels (36.23% of all marmoset indels) were reciprocally lifted over to human. The RECOVER_SWAPPED_REF_ALT = true option in Picard LiftoverVcf only works for SNVs and does not work for indels.

The variants projected onto the human genome were then annotated for predicted pathogenicity using CADD (v1.6) [27]. Stop gained and frameshift variants were annotated with a probability of being loss-of-function intolerant (pLI) using scores based on the number of observed versus expected protein-truncating variants that have been calculated across the human gnomAD dataset [28].

### 2.11. Marmoset SNVs Orthologous to ClinVar SNVs

ClinVar [16] is a public database that collects and shares information about genetic variants and their association with human health and disease. We identified marmoset SNVs orthologous to ClinVar SNVs and noted the associated diseases. We emphasized SNVs that cause the same consequence in the same annotated gene of marmosets and humans since those marmoset SNVs are more likely to be directly relevant to the disease. We also emphasized SNVs with a ClinVar significance of pathogenic, pathogenic/likely pathogenic, or likely pathogenic due to the increased likelihood that those SNVs are functionally significant.

### 2.12. Neurodevelopmental Gene Variants

We identified variants in marmoset genes orthologous to the human genes identified in studies by Satterstrom et al. [29] and Coe et al. [30]. Satterstrom et al. [29] analyzed 35,584 samples including 11,986 with autism spectrum disorder (ASD) and identified 102 risk genes with a false discovery rate of less than 0.1. Among these genes, 49 showed higher disruptive de novo variant frequencies in individuals with severe neurodevelopmental delay and 53 exhibited higher frequencies in individuals with ASD. Coe et al. [30] used de novo mutation (DNM) data from 10,927 individuals with developmental delay and autism and identified 253 candidate neurodevelopmental disease genes with an excess of missense and/or likely gene-disruptive mutations.

### 2.13. Alzheimer’s Disease Gene Variants

We identified variants in marmoset genes orthologous to human genes implicated in Alzheimer’s disease by Stage et al. [31] and Bellenguez et al. [32]. Stage et al. [31] performed genetic associations between the top 20 Alzheimer’s disease (AD) risk alleles and amyloid deposition and neurodegeneration. Bellenguez et al. [32] examined AD and related dementia (ADD) in a meta-analysis of genome-wide association studies (39,106 clinically AD-diagnosed cases, 46,828 proxy-ADD cases, and 401,577 controls) and followed up the most promising signals in 25,392 independent AD cases and 276,086 controls. They reported 75 risk loci for ADD, including 42 novel ones.

### 2.14. Callitrichine Size and Reproduction-Related Genes

We previously identified callitrichine base substitutions possibly fixed in marmoset species and affecting genes likely to have a functional role in the evolution of reduced body size or the unusual reproductive traits characteristic of marmosets and callitrichines in general [2]. We used the current marmoset SNV data to determine which of those proposed fixed base substitutions are actually polymorphic in marmosets.

## 3. Results

### 3.1. Genome Assembly and Gene Annotation

We generated a high-quality genome assembly for the common marmoset using long-read PacBio sequencing data along with several types of mapping data. Our assembly, Callithrix_jacchus_cj1700_1.1, is comparable in all metrics of assembly contiguity to the current marmoset reference mCalJa1.2.pat.X (Table S2), but some improvements were observed. Our version has a total length of 2.859 Gbp, with contig N50 length and scaffold N50 length of 25 Mbp and 98 Mbp, respectively. However, we report a larger reduction (1.8-fold or 1335 vs. 2398 contigs) in assembly gaps as indirectly measured by total contigs (Table S2). The assembly completeness assessed using mapped same-species transcripts from GenBank (*n* = 1624) was 98.8%. Further assessment of gene representation using BUSCO (13,780 primate genes) showed a high level of completeness at 98.2% (Table S3). When comparing assembled versions of multiple marmoset genomes for annotated genes, we again observe similar numbers of protein-coding and noncoding genes and mRNAs (Table S4).

### 3.2. Single Nucleotide Variants

We identified a total of 19,137,198 SNVs across the 84 marmosets, including 378,827 multiallelic SNVs and 2,078,752 singletons (Table S5). The transition/transversion ratio of the SNVs was 1.92. We found an average of 5,427,648 SNVs per individual. Genetic consequences include 74,088 missense and 1366 stop gained SNVs with potential functional significance (Table S5). All of the variants reported here were submitted to the Marmoset Coordinating Center and its online database (https://mcc.ohsu.edu, accessed on 28 November 2023).

### 3.3. Indel Variants

We identified a total of 2,816,760 small indels from 1–6 bp in size across the 84 marmosets, including 541,631 multiallelic indels and 384,507 singletons (Table S6). There was an average of 852,024 indels per individual. These include potential changes in protein-coding genes: 2773 frameshift indels, 750 inframe insertions, and 876 inframe deletions with potential functional significance (Table S6).

### 3.4. Population Genetics

**Figure 1:**
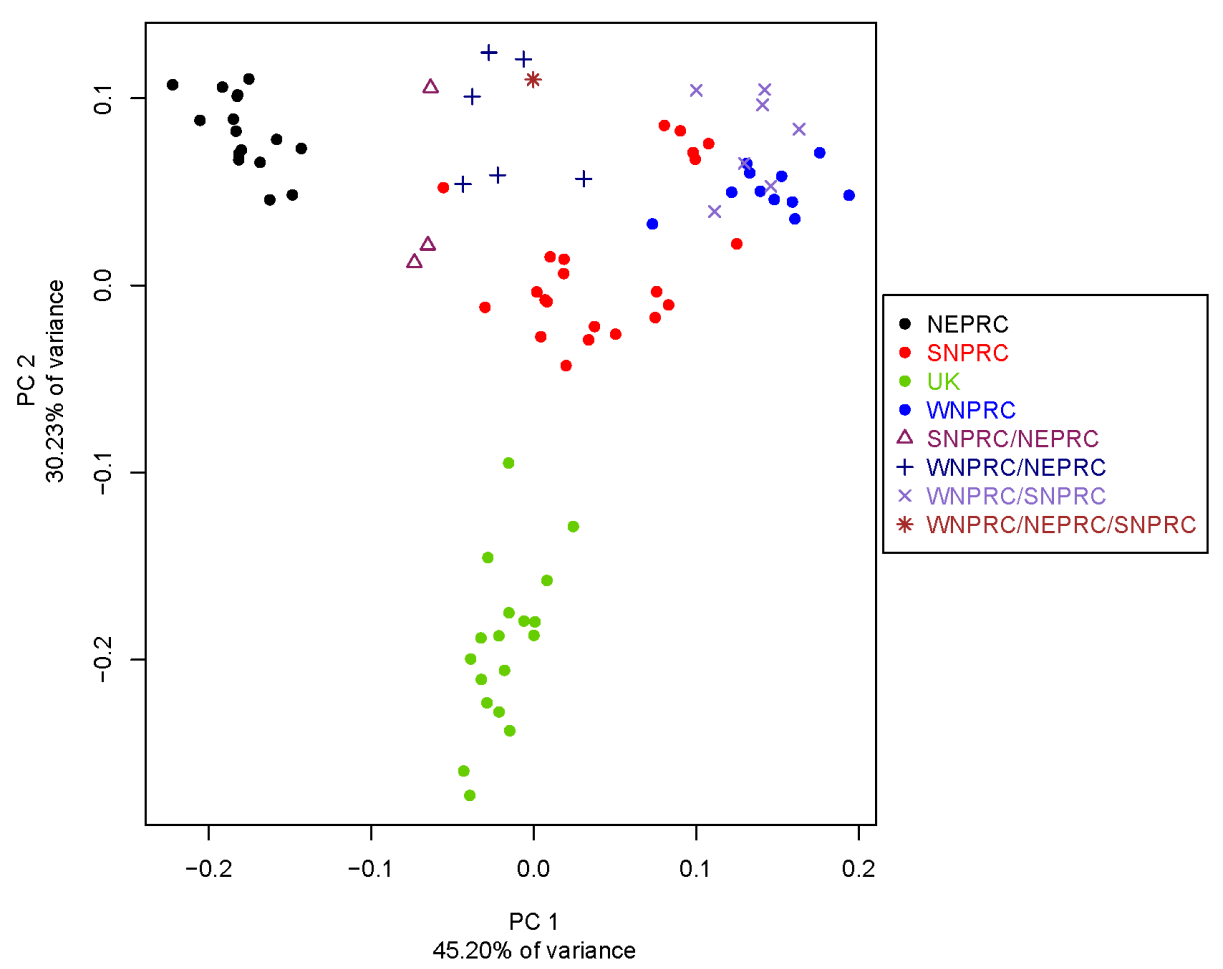
PCA plot of 84 marmosets originating from four research centers or born from parents originating from different centers (e.g., SNPRC x NEPRC). NEPRC: New England Primate Research Center; SNPRC: Southwest National Primate Research Center; UK: Defence Science and Technology Laboratory, United Kingdom; and WNPRC: Wisconsin National Primate Research Center.

We generated a principal components analysis (PCA) plot for the 84 marmosets based on autosomal SNVs (Figure 1). The NEPRC population forms a discrete cluster separated on PC1, which explains 45.20% of the variance. The UK population forms a discrete cluster separated by PC2, which explains 30.23% of the variance. The WNPRC and SNPRC populations along with known crosses, either between those two colonies or between one of them and the UK or NEPRC colonies, form a third less well-defined cluster. ADMIXTURE analyses identified the best-fitting model as K = 3 (CV error = 0.26717) (Figure S1). In the ADMIXTURE analysis, the UK individuals form a unique population and all NEPRC individuals fall within a single, different population. Individuals from WNPRC, SNPRC, and the known crosses among those two centers or NEPRC individuals tend to cluster as one extended group showing varying degrees of admixture and individual variation.

### 3.5. Genome Diversity

**Figure 2:**
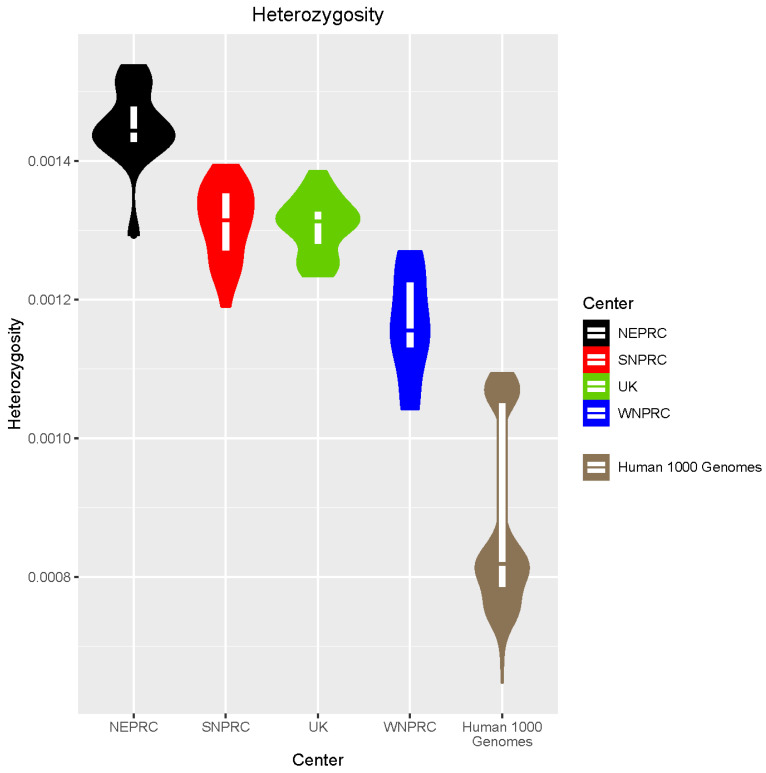
Genome-wide autosomal heterozygosity of marmosets by center. Heterozygosity was calculated as (autosomal heterozygous SNV calls)/(ungapped autosomal assembly length − missing SNV calls) for each individual animal and then plotted separately for each center. WNPRC shows relatively lower heterozygosity suggesting reduced diversity, but humans show even less diversity based on 1000 Genomes data.

The average heterozygosity across the 84 marmoset samples is 0.00133 (Figure 2), which is consistent with other reported heterozygosity estimates for this species [13]. WNPRC shows the lowest average heterozygosity (0.00116), which is significantly (Welch *t*-test *p* = 2.341 × 10^−6^) lower than the other colonies. However, even the WNPRC individuals show significantly more heterozygosity than humans (Welch *t*-test *p* = 1.281 × 10^−7^), which have an average heterozygosity of 0.00087 based on the 1000 Genomes Project [33]. The average marmoset inbreeding coefficient (F) across all samples is −0.02894 (Figure S2). WNPRC has the highest inbreeding coefficient (0.0875), which is significantly higher compared with the other colonies (Welch *t*-test *p* = 4.397 × 10^−6^). However, even the WNPRC individuals show a significantly lower inbreeding coefficient (Welch *t*-test *p* = 0.01532) compared with humans with non-African ancestry (average F = 0.13641) from the 1000 Genomes Project. We identified runs of homozygosity (ROH) in the marmosets and the average fraction of the genome demonstrating ROH (Froh) was 0.0235 (Figures S3 and S4).

### 3.6. Marmoset SNVs Orthologous to ClinVar SNVs

ClinVar [16] is a database of human genetic information created and managed by the National Center for Biotechnology Information (https://www.ncbi.nlm.nih.gov/clinvar/, accessed on 28 November 2023). This website provides access to extensive lists of human genetic variants and their clinical or phenotypic significance. A total of 11,872,309 marmoset SNVs (62.04% of total SNVs) reciprocally lifted over to the human genome. Among those, 5561 were orthologous to ClinVar SNVs and involved the same alleles. In 4956 cases (89.12%), a particular SNV mapped to the same annotated gene in both marmoset and human and the marmoset predicted functional consequence was the same as one of those that occur among the different human isoforms. The ClinVar clinical significance was pathogenic and/or likely pathogenic for 37 of the 5561 orthologous SNVs including 27 with the same consequence in the same annotated gene in marmoset and human (Table S7). There were 1456 SNVs orthologous between marmoset and human with a ClinVar clinical significance of “uncertain significance”. This includes 1274 with the same consequence and gene annotation. Additionally, there were 419 SNVs with conflicting ClinVar interpretations of pathogenicity including 367 with the same consequence and gene annotation in marmoset and human.

Diseases represented among the orthologous ClinVar SNVs with the same consequence in the same annotated gene of marmoset and human include congenital heart disease and maturity-onset diabetes mellitus in young (Table 1). Additional details regarding these SNVs together with information about SNVs with other ClinVar significance designations are shown in the Supplementary Materials, Table S8.

### 3.7. Neurodevelopmental Gene Variants

We examined SNVs in marmoset genes orthologous to 297 human genes implicated in autism and neurodevelopmental disorders [29,30]. In humans, de novo deleterious variants in these genes are thought to be dominant and have a large effect, but mouse models often fail to replicate the complexity of neurobehavioral features of human disease [30]. We filtered the SNVs to include only those with a CADD PHRED score >20. A CADD PHRED score of that magnitude predicts that the change is among the 1% most functional changes that can occur in the human genome. We identified 277 missense SNVs and 3 stop gained SNVs across the neurodevelopmental genes (Table S9). The scores for the probability of being loss-of-function intolerant (pLI) are based on the number of observed versus expected protein-truncating variants and have been calculated across the human gnomAD dataset [28]. The closer the pLI score is to 1, the more intolerant to stop gained SNVs the gene is predicted to be. The stop gained SNVs occurred in *HDLBP* (CADD 38; pLI 1.00), *CDK13* (CADD 36; pLI 0.91), and *VEZF1* (CADD 35; pLI 0.99). The high CADD PHRED score and pLI scores approaching 1 suggest these SNVs are likely to have functional consequences.

We also examined small indels in the autism and neurodevelopmental disorder genes. There are 11 indels with a CADD PHRED score >20 that affect the encoded protein. These include eight frameshifts, two inframe deletions, and one inframe insertion (Table S10). Among the frameshift indels, four occur in genes with a pLI score > 0.9: *SETD5* (CADD 33; pLI 1.00), *VEZF1* (CADD 27.9; pLI 0.99), *ARID1B* (CADD 25; pLI 1.00), and *VEZF1* (CADD 24.4; pLI 0.99). The two *VEZF1* frameshift indels occur downstream from the *VEZF1* stop gained SNV in a heterozygous state in the same 24 individuals. This suggests that all of these variants are in linkage disequilibrium and occur in the same haplotype. The introduction of the stop gained SNV may have reduced selective constraints on the downstream region, thereby reducing the functional consequences of these specific frameshift indels.

### 3.8. Alzheimer’s Disease Gene Variants

We examined SNVs in marmoset genes orthologous to 83 human genes implicated in Alzheimer’s disease [31,32]. We also filtered these SNVs to include only those with a CADD PHRED score >20. We identified 91 missense SNVs and one stop gained SNV across the Alzheimer’s disease genes (Table S11). The stop gained SNV occurs in *ABCA7* and has a CADD PHRED score of 48, which strongly suggests it has a functional consequence.

We also examined small indels in the Alzheimer’s disease genes. There are four indels with a CADD PHRED score >20 that affect the expressed protein, including two frameshifts (*ABCA7*, CADD 25.2; *LIME1* CADD 23.6), 1 inframe deletion (*PTK2B*, CADD 22), and 1 splice acceptor variant (*EPHA1*, CADD 35) (Table S12). None of the frameshift indels occur in genes with a pLI score >0.9.

### 3.9. Callitrichine Size and Reproduction-Related Genes

Prior studies identified a small number of genes as possibly playing meaningful roles in the evolution of reduced body size and the unusual reproductive traits characteristic of marmosets and callitrichines in general [2]. In those analyses, we presented evidence that *WFIKKN1, GDF9, BMP4,* and *BMP15* may influence the process of frequent twinning in marmosets. That early study included sequence data from only a small number of marmoset individuals and, therefore, we could not extensively screen for within-species variation affecting the amino acid changes suggested to underlie the functional changes in these proteins. Now in the present study, we find that only two out of the 219 base substitutions that were implicated as potentially having evolutionary consequences display polymorphism (Table S13). Thus, most of the changes identified in that early study remain candidates as substitutions that resulted from selection for adaptations altering reproductive biology.

### 3.10. Marmoset Variants UCSC Genome Browser Track Hub

The marmoset variants we identified can be visualized through a UCSC Genome Browser track hub [34] (https://genome.ucsc.edu/cgi-bin/hgTracks?db=calJac4&position=chr13%3A7334184-7334684&hubUrl=ftp://ftp.hgsc.bcm.edu/ucscHub/marmosetVariants/hub.txt, accessed on 28 November 2023). The track hub includes SNV and indel tracks on both our new marmoset Callithrix_jacchus_cj1700_1.1 assembly and tracks of the variants reciprocally lifted over to the human hg38 assembly. The variants are annotated with relevant information such as variant consequence, population allele frequency, and genotypes of individual samples. This resource serves as a valuable tool for comparative genomics, evolutionary biology, and disease association studies, enabling researchers to gain a better understanding of marmoset genetic diversity and variation. Data on variants will also be submitted to the Marmoset Coordinating Center (https://mcc.ohsu.edu/, accessed on 28 November 2023).

## 4. Discussion

The genome assembly, annotated gene set, and initial variant collection for the common marmoset presented in this study will serve as valuable resources for biomedical and evolutionary researchers using this model organism. The improved assembly will aid in gene annotation and identification of genetic variants associated with traits of interest. The large number of SNVs identified in this cohort of animals can be used for population genetics and other genomic comparisons designed to better understand the genetic diversity and structure of different marmoset populations. Increased information about genetic variation among individual marmosets in research colonies will provide many new opportunities for investigation. For example, the missense or stop-gain variants we identified in genes associated in humans with Alzheimer’s disease, neurodevelopmental disorders, or other clinical conditions may in some cases be useful for the development of naturally occurring marmoset models of human disease. This approach has been successful with regard to rhesus macaque models of human disease [14,35,36]. One recurrent problem in the management of marmoset colonies is a high rate of early neonatal mortality [37]. Having whole genome sequence data for a sufficient number of breeding marmosets, surviving offspring and deceased offspring would facilitate genetic analyses that might identify specific genetic factors influencing early infant survival.

In addition, an increasing number of researchers are using CRISPR/Cas9 and other technologies to conduct genetic engineering studies using marmosets, with the goal of generating custom models of human genetic disease [3]. Access to whole genome sequence data for prospective subjects of these genetic manipulation experiments will allow investigators to choose the optimal study animals for their work. For example, researchers intending to generate CRISPR/Cas9 mutations that affect genes associated with autism in humans could ensure the maximum value and impact of their new model by ensuring that the animals used to initiate the manipulation and establish the genetically modified breeding line do not carry predicted damaging mutations in other genes also associated with autism or neurodevelopment. Finally, natural variation exists among marmosets in a number of phenotypes relevant to biomedical research, including obesity [38] and behavioral responses to various experimental paradigms [39,40,41]. Extensive genotyping of marmoset colonies using whole genome sequence data would facilitate genetic association studies that could potentially identify genetic differences among animals that contribute to this phenotypic variation.

Given the continuing reductions in the cost of whole genome sequencing, there is a tremendous opportunity to develop detailed and extensive information regarding genetic diversity among the common marmosets in US research colonies. The presence of hematopoietic chimerism in marmosets complicates the production of individual specific genome sequences since blood samples from individual marmosets will contain cells and genomic DNA inherited by their co-twin and transferred to the subject animal during embryogenesis [20]. The use of fibroblast cell lines as the source of DNA samples for sequencing solves this problem and also provides other advantages such as a permanent source of banked cells suitable for subsequent production of induced pluripotent stem cells or organoids [42]. But of course, this does add cost. Low-coverage genome sequencing using hair follicle DNA has been performed and can generate estimates of kinship among individuals [43] (Cole et al.; doi.org/10.1101/2023.06.22.545969), but suffers in comparison to deep (30x) whole genome coverage due to the inability of low-coverage sequencing to accurately estimate individual heterozygosity, detect more than a fraction of the SNVs present in a given animal, or fully characterize the potential disease-related mutations carried by any given study population. Given the significance of marmosets for a wide range of biomedical studies, deep whole genome sequencing is warranted as a means of providing researchers with complete and valuable genetic information for their study subjects.

## 5. Conclusions

We generated a new genome assembly and identified more than 19 million SNVs, including 74,088 missense variants, among 84 common marmosets. The new assembly has improved contiguity and completeness compared with previous assemblies, and the variant catalog provides a valuable resource for researchers using this model organism for biomedical research. While significantly improved relative to earlier versions of the marmoset genome, this assembly is not yet complete. Advances in long-read sequencing over the next few years will allow for the development of a telomere-to-telomere assembly where segmental duplications, centromeres, and acrocentric short arms are fully resolved [44,45]. Nevertheless, the availability of this high-quality resource that captures most orthologs of human genes will facilitate experiments that can be translated to issues of human health and will also enhance our understanding of the biology of the common marmoset.

## Figures and Tables

**Table 1:** Marmoset SNVs orthologous to ClinVar SNVs. These orthologous SNVs cause the same consequence in the same annotated gene of marmoset and human and have a ClinVar significance of pathogenic, pathogenic/likely pathogenic, or likely pathogenic. We also list the functional consequences reported by the Variant Effect Predictor (VEP) and the allele frequency observed across the 84 study animals.

Marmoset SNV ID	dbSNP	Gene	Marmoset VEP Consequence	Allele Freq.	ClinVar Disease
**Pathogenic**					
13:7334434:C:T	rs864321699	GATA4	Missense	0.0179	Congenital heart disease
15:3504507:T:C	rs577069249	CCDC39	Intron	0.1369	Primary ciliary dyskinesia
16:51588847:C:T	rs994355873	RECQL4	Stop gained	0.0060	Baller–Gerold syndrome
19:10341384:C:T	rs770329105	USH2A	Missense	0.0833	Usher syndrome type 2A
22:39820446:C:T	rs80356463	SIX5	Missense	0.0060	Branchiootorenal syndrome 2
2:44720125:C:A	rs191068215	SPINK1	5 prime UTR	0.0119	Tropical pancreatitis
4:17947215:G:A	rs104893960	GCM2	Missense	0.0357	Hypoparathyroidism, familial isolated, 2
5:96794138:G:A	rs764561297	HNF1B	Synonymous	0.0238	Maturity onset diabetes mellitus in young
7:149491445:G:A	rs606231300	SLC16A1	Stop gained	0.2321	Monocarboxylate transporter 1 deficiency, autosomal dominant
9:68087733:C:T	rs1942608745	KMT2D	Stop gained	0.3988	Kabuki syndrome
9:68088117:C:T	rs1942584412	KMT2D	Missense	0.3988	Kabuki syndrome
**Pathogenic/Likely Pathogenic**					
14:36823751:C:T	rs1057521141	DYSF	Missense	0.0238	Autosomal recessive limb-girdle muscular dystrophy type 2B/qualitative or quantitative defects of dysferlin
5:65779382:G:A	rs104894553	ASPA	Missense	0.5000	Spongy degeneration of the central nervous system/mild Canavan disease/inborn genetic diseases
9:68087587:G:A	rs886041404	KMT2D	Missense	0.3976	Kabuki syndrome
9:68087980:G:A	rs267607237	KMT2D	Missense	0.3988	Kabuki syndrome
**Likely Pathogenic**					
10:107534744:C:T	rs751122392	IFT43	Intron	0.0179	Not provided
10:42430818:C:T	rs979186313	KNL1	Synonymous	0.7917	Microcephaly 4, primary, autosomal recessive
11:5831070:A:G	rs864622543	ATM	Intron	0.4702	Ataxia–telangiectasia syndrome/hereditary cancer-predisposing syndrome
18:26568817:T:C	rs118203910	F5	Missense	0.0238	Factor V deficiency
2:1601055:G:T	rs200853731	ASL	Missense	0.0119	Argininosuccinate lyase deficiency
4:46330371:C:T	rs2113877345	TREM2	Splice acceptor	0.0119	Not provided
4:57040110:C:A	rs2128209217	PKHD1	Splice donor	0.7440	Autosomal recessive polycystic kidney disease
5:10531420:T:C	rs576243101	PROKR2	Missense	0.3155	Not provided
5:36862718:G:A	rs35187177	SGK2	Missense	0.0179	Colorectal cancer
5:5037801:C:T	rs748725549	XKR7	Missense	0.0119	Moyamoya angiopathy
9:68087755:G:T	rs2120361380	KMT2D	Missense	0.3988	Kabuki syndrome 1
9:90628336:C:T	rs1381940328	CEP290	Intron	0.1905	Leber congenital amaurosis 10

## Data Availability

All data will be available at NCBI SRA under NCBI BioProjects PRJNA566173 and PRJNA955237, and also at https://mcc.ohsu.edu/.

## Supplementary Materials

The following supporting information can be downloaded at: https://www.mdpi.com/article/10.3390/genes14122185/s1, Figure S1: ADMIXTURE plot of 84 marmosets originating from or born from parents from four research centers; Figure S2: Inbreeding coefficient estimates of marmosets by center; Figure S3: F_ROH_ estimates of marmosets by center; Figure S4: F_ROH_ estimates of marmosets by center for each chromosome; Table S1: Marmoset Samples Sequenced; Table S2: Representative assembly metrics for sequenced marmoset genomes; Table S3: BUSCO scores for multiple assembled marmoset genomes; Table S4: Representative gene annotation metrics for sequenced marmoset genomes; Table S5: SNV Summary Stats; Table S6: Indel Summary Stats; Table S7: ClinVar Marmoset Stats; Table S8: ClinVar Details; Table S9: Neuro Genes SNVs; Table S10: Neuro Genes Indels; Table S11: Alzheimer’s SNVs; Table S12: Alzheimer’s Indels; Table S13: Twinning.
