# Supplementary material for: Whole Genome Analysis of SNV and Indel Polymorphism in Common Marmosets (Callithrix jacchus)
Source: Genes (Basel). 2023 Dec 7;14(12):2185. doi: 10.3390/genes14122185 (PMC10742769; doi:10.3390/genes14122185)
Supplement: Supplementary file 1 [file genes-14-02185-s001.zip › marmoset.WGSpaper.supplemental.pdf]

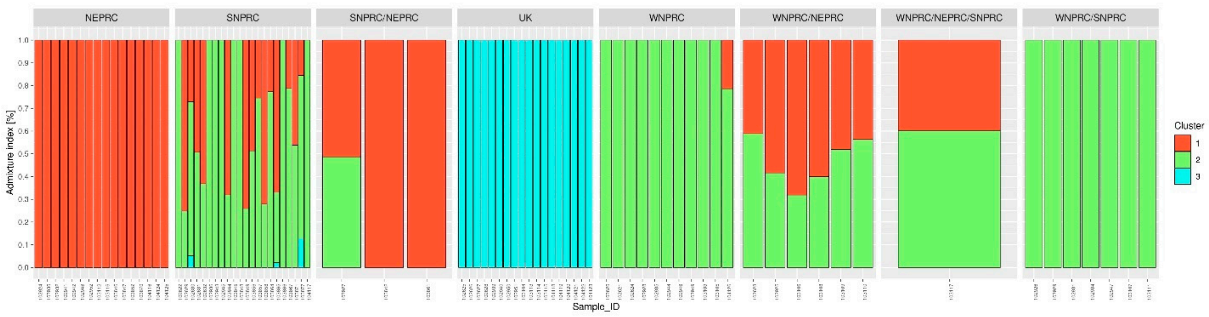

Figure S1. ADMIXTURE plot of 84 marmosets originating from or born from parents from four research centers. The ADMIXTURE cross-validation procedure identified the best fitting model as K=3 (CV error = 0.26717).

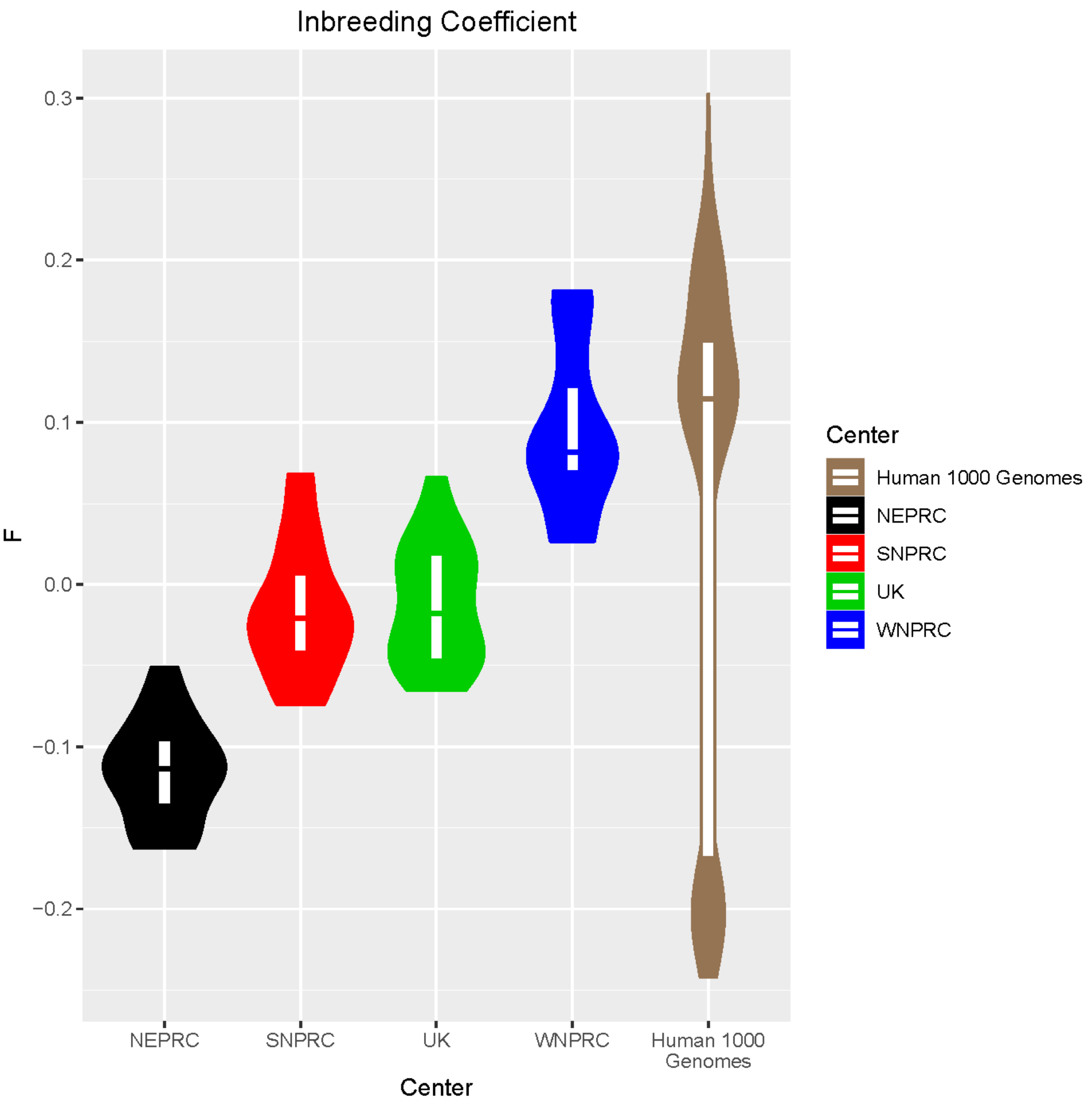

Figure S2. **Inbreeding coefficient estimates of marmosets by center.** Method-of-moments F inbreeding coefficient estimates were calculated with PLINK as (observed homozygous SNVs - expected homozygous SNVs) / (total called SNVs - expected homozygous SNVs) for each individual and then plotted separately for each center. WNPRC shows relatively higher inbreeding coefficients suggesting reduced diversity.

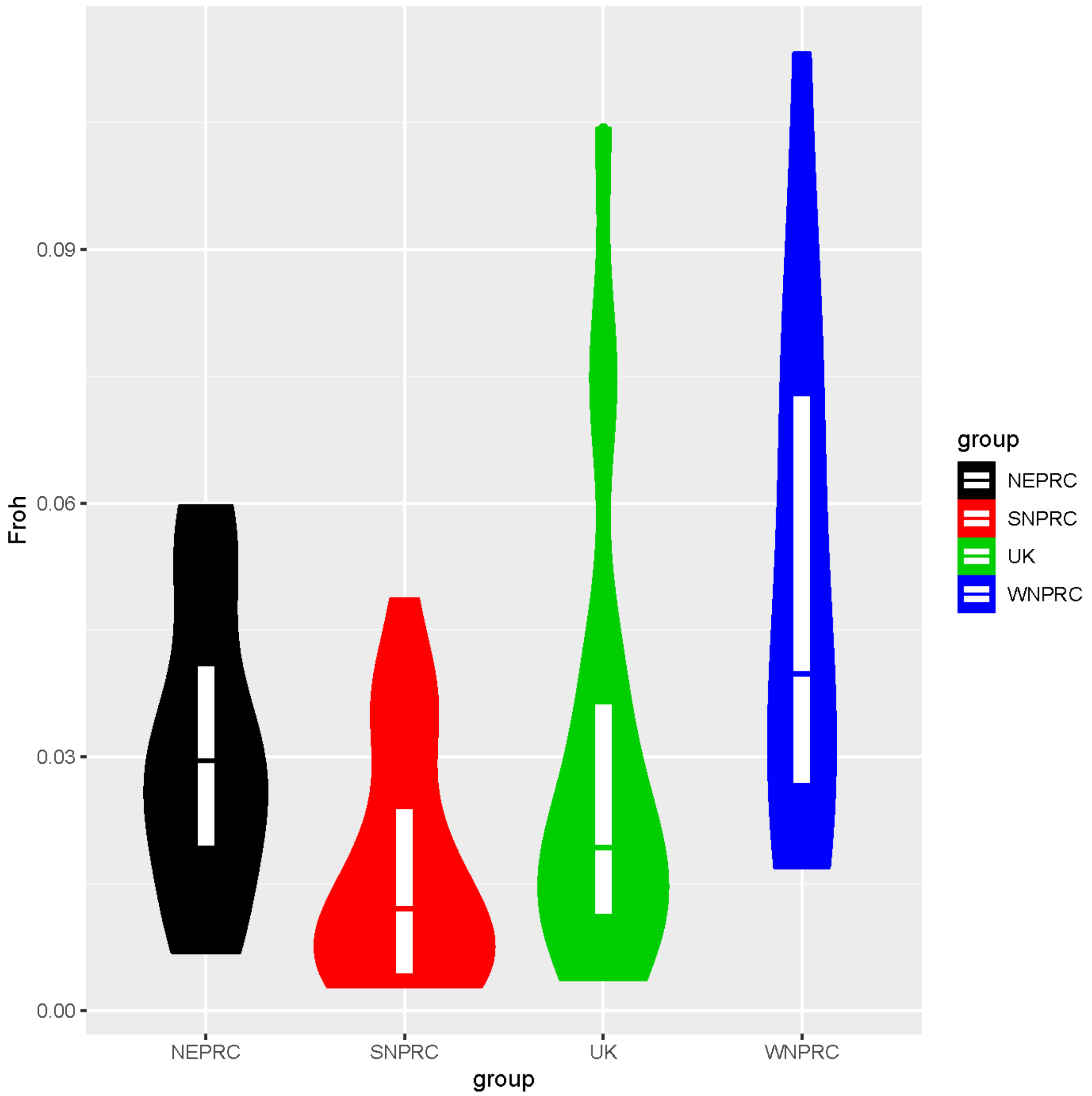

Figure S3. **F_{ROH} estimates of marmosets by center**. ROHs were calculated by PLINK and F_{ROH} was calculated as the proportion of runs of homozygosity (ROH) in the autosomal genome per individual and then plotted separately for each center. WNPRC shows relatively higher F_{ROH} suggesting reduced diversity.

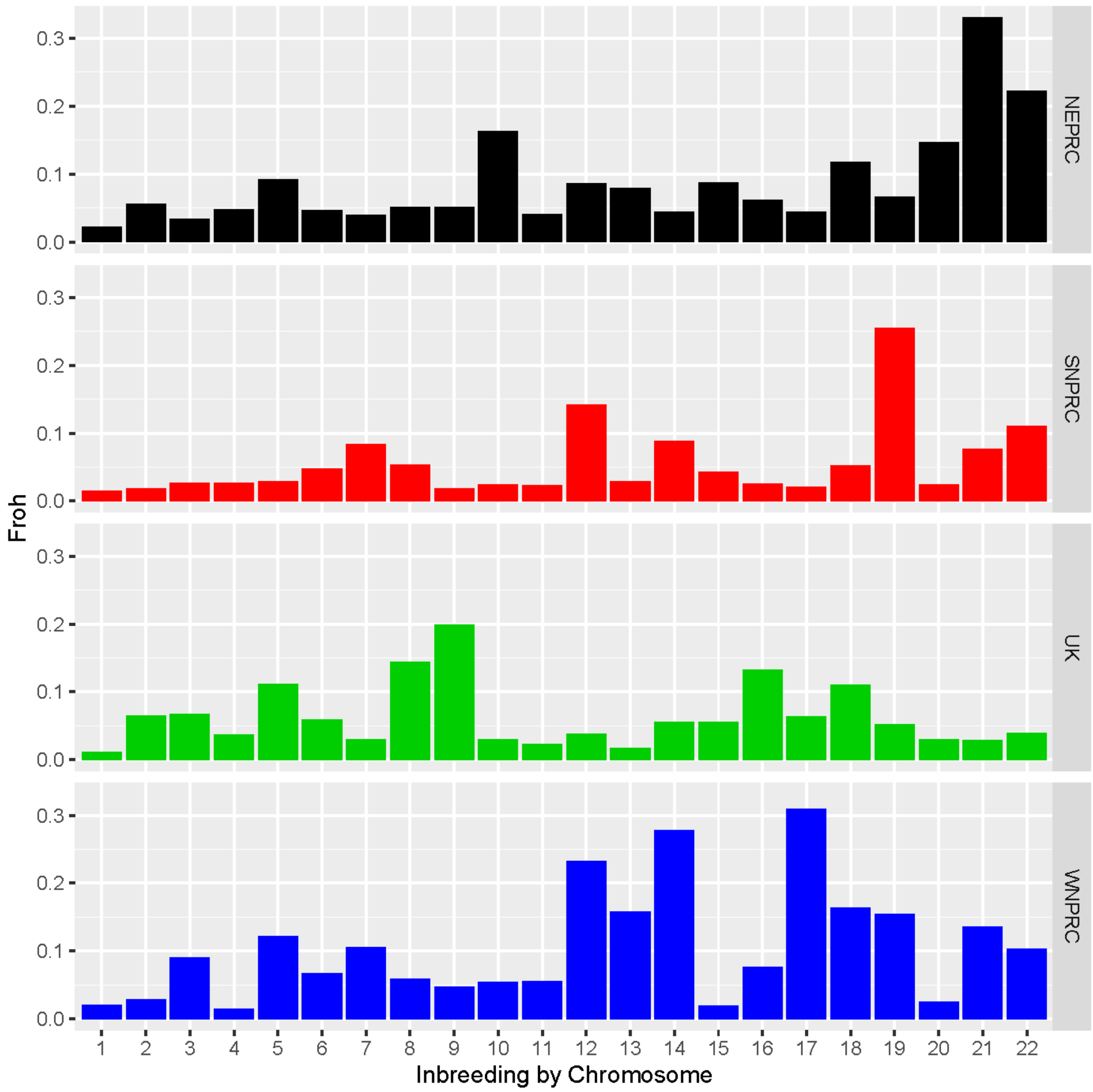

Figure S4. **F_{ROH} estimates of marmosets by center for each chromosome**. ROHs were calculated by PLINK and F_{ROH} was calculated as the proportion of runs of homozygosity (ROH) in each chromosome per individual and then plotted separately for each center. WNPRC animals show a relatively higher F_{ROH} in most chromosomes suggesting reduced diversity.
